# MiR-223-3p promotes cell proliferation and metastasis by downregulating SLC4A4 in clear cell renal cell carcinoma

**DOI:** 10.18632/aging.101763

**Published:** 2019-01-22

**Authors:** Wen Xiao, Xuegang Wang, Tao Wang, Jinchun Xing

**Affiliations:** ^1^ Department of Urology, The First Affiliated Hospital of Xiamen University, Xiamen, Fujian, China; ^2^ Center of Diagnosis and Treatment of Urinary System Diseases, The First Affiliated Hospital of Xiamen University, Xiamen, Fujian, China; ^3^ The Key Laboratory of Urinary Tract Tumors and Calculi of Xiamen City, The First Affiliated Hospital of Xiamen University, Xiamen, Fujian, China

**Keywords:** miR-223-3p, ccRCC, oncomiRNA, SLC4A4, metastasis

## Abstract

MicroRNAs (miRNAs) are known to affect the occurrence and progression of cancer. We therefore evaluated the involvement of miR-223-3p in renal cell cancer. MiR-223-3p was highly expressed in clear cell renal cell cancer tissues. Clear cell renal cell cancer patients with higher miR-223-3p expression had higher tumor stages and grades and poorer prognoses. In renal cancer cells, overexpression of miR-223-3p enhanced cell proliferation and metastasis, while inhibition of miR-223-3p reduced the malignant capacity of the cells. MiR-223-3p was found to bind directly to solute carrier family 4, member 4 (*SLC4A4*) mRNA, thereby reducing SLC4A4 mRNA and protein expression. SLC4A4 overexpression restrained cell proliferation and metastasis by suppressing Kirsten rat sarcoma viral oncogene (KRAS) expression in renal cancer cells. *SLC4A4* expression correlated negatively with miR-223-3p expression in patient samples. Given that miR-223-3p suppressed the SLC4A4/KRAS axis, miR-223-3p gene therapy could be an effective treatment for renal cancer.

## INTRODUCTION

Kidney cancer accounts for 3.77% of adult malignancies, and is a common lethal urological malignancy. In 2018, about 65,340 new kidney cancer cases and 14,970 deaths were estimated in the United States [1], and about 403,262 new kidney cancer cases (2.2% of all cancers) and 175,098 deaths (1.8% of all cancers) were reported worldwide [2]. Renal cell carcinoma (RCC) accounts for more than 90% of kidney cancer cases. The disease originates from the renal epithelium and encompasses more than 10 histological and molecular subtypes [3]. The incidence of RCC has increased gradually in the past two decades, and up to 30% of patients with a primary diagnosis of RCC will experience cancer metastasis [4, 5].

Clear cell RCC (ccRCC) is the most common RCC subtype, and has the highest rate of mortality, invasion and metastasis [6]. Given the extreme lethality of ccRCC, early diagnosis is critical for its treatment. Thus, it would be significant to determine the molecular mechanisms responsible for the genesis and development of ccRCC.

MicroRNAs (miRNAs), first discovered in 1993 [7], are small, non-coding RNA molecules that contribute to multiple cellular processes, including cancer cell proliferation [8, 9], apoptosis [10], metastasis [11, 12], radiation sensitivity [13] and chemosensitivity [14]. MiRNAs may function as oncogenes (oncomiRNAs) or tumor suppressors by downregulating the transcription of various genes [15, 16]. Mature miRNAs bind to the mRNA of target genes to degrade them and inhibit gene translation [17–19]. Recently, several miRNAs have been identified as cancer biomarkers associated with disease progression, including miR-31-5p, miR-144-3p and miR-129-3p [20–22].

MiR-223-3p is frequently upregulated in tumors from different organs and tissues, such as hepatocellular carcinoma [23], lung cancer [24], gastric cancer [25] and prostate cancer [26]. Nevertheless, to the best of our knowledge, the involvement of miR-223-3p in ccRCC has not yet been investigated. Therefore, in the present study, we systematically examined the function of miR-223-3p in ccRCC, including its expression in ccRCC tissues, its association with the pathological tumor (T) stage, grade and prognosis, and its effects on RCC cell proliferation, migration and invasion *in vitro*. We also determined its target mRNA molecule and its downstream effects on gene expression in RCC.

## RESULTS

### MiR-223-3p is significantly upregulated and associated with a poor prognosis in ccRCC patients in TCGA-KIRC

**Figure 1:**
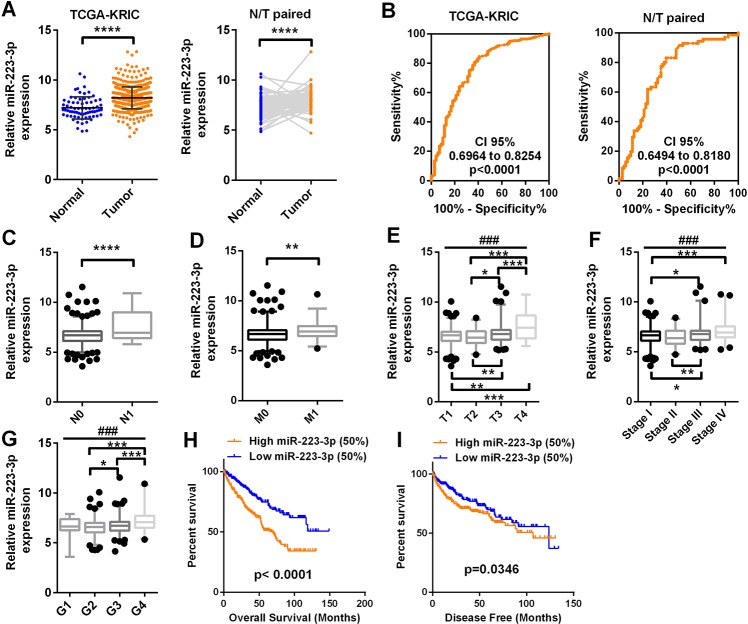
**MiR-223-3p expression is upregulated in ccRCC and predicts a poor prognosis.** MiR-223-3p levels in 71 normal tissues and 506 ccRCC tissues were downloaded from the dataset of TCGA-KIRC. The levels of miR-223-3p were compared according to different clinicopathological parameters: (**A**) cancer versus para-cancer, and cancer versus paired para-cancer. (**B**) The ROC curve displays that miR-223-3p could effectively distinguish ccRCC from para-cancer and paired para-cancer tissues, with AUCs of 0.7609, CI 95% (0.6964 to 0.8254) (p < 0.0001) and 0.7337, CI 95% (0.6494 to 0.8180) (p < 0.0001), respectively. (**C**–**I**) MiR-223-3p expression was analyzed in ccRCC patients according to (**C**) lymph node metastasis, (**D**) distant metastasis, (**E**) T stage, (**F**) TNM stage, (**G**) grade, (**H**) OS and (**I**) DFS. Data are shown as the mean ± SEM. * p < 0.05; ** p < 0.01; *** p < 0.001; ### p < 0.001.

Gene expression data from ccRCC patients were obtained from The Cancer Genome Atlas (TCGA) ccRCC dataset (TCGA-KIRC). Detailed clinicopathological information on these patients is presented in Table 1. The relative expression of miR-223-3p, shown in log2 (FPKM+1) form, ranged from 4.85 to 10.62 units in normal tissues and from 3.59 to 10.92 units in tumor tissues. MiR-223-3p expression was significantly higher in ccRCC tissues than in the corresponding non-cancerous tissues. Similar results were obtained from 71 paired ccRCC tissues and corresponding non-cancerous tissues (Figure 1A). Receiver operator characteristic (ROC) curve analysis demonstrated that miR-223-3p could sufficiently discriminate ccRCC tissues from normal tissues (area under the curve [AUC]: 0.7609, 95% confidence interval: 0.6964 - 0.8254) and from paired normal tissues (AUC: 0.7337, 95% confidence interval: 0.6494 - 0.8180) (Figure 1B). We also screened the database for a classic oncomiRNA (miR-21) and several miRNAs from recently published articles (miR-452, miR-543 and miR-708). We found that miR-223-3p was less sensitive than miR-21, but more sensitive than miR-708 (Supplementary Figure 1).

**Table 1:** Correlations of miR-223-3p and SLC4A4 levels in tissues and clinicopathological parameters of ccRCC patients **Correlation of miR-223-3p and SLC4A4 expression with clinicopathological parameters.**

	miR-223-3p expression			SLC4A4 mRNA expression		
Parameter	Low (n=250)	High (n=250)	P value	Low (n=250)	High (n=250)	P value
Age(years)			0.283			0.795
<=60	132	119		130	121	
>60	118	131		120	129	
Sex			0.259			0.090
Male	170	157		173	154	
female	80	93		77	96	
T stage			0.041			0.354
T1+T2	169	146		152	163	
T3+T4	81	104		98	87	
N stage			0.417			0.173
N0+ NX	245	241		240	246	
N1	5	9		10	4	
M stage			0.013			0.137
M0+ MX	222	201		205	218	
M1	28	49		45	32	
Grade			0.002			0.472
G1+G2	131	96		109	118	
G3+G4	119	154		141	132	
TNM stage			0.018			0.202
I+II	162	135		141	156	
III+IV	88	115		109	94	

T-test analysis demonstrated that miR-223-3p expression was higher in patients with lymph node metastases than in those without (Figure 1C). High miR-223-3p expression was also observed in patients with distant metastases (Figure 1D). MiR-223-3p expression was significantly higher in T stages III and IV than in T stages I and II, and was greater in T stage IV than in T stage III (Figure 1E). Higher miR-223-3p levels were associated with more advanced pathological TNM stages (T stage, lymphatic metastasis and distant metastasis grouping) and grades in ccRCC patients (Figure 1F and 1G).

Kaplan-Meier survival analysis was used to determine the correlation of miR-223-3p expression with the overall survival (OS) and disease-free survival (DFS) of ccRCC patients. Patients from TCGA-KIRC were divided into two groups based on the median miR-223-3p level. Patients with higher miR-223-3p expression (> 6.7 units) exhibited shorter OS (Figure 1H, p < 0.0001) and DFS (Figure 1I, p = 0.0346). The expression of miR-223-3p was then compared between patients with longer and shorter OS and DFS (OS-Good and OS-Poor, DFS-Good and DFS-Poor). High miR-223-3p expression indicated a poor prognosis (Supplementary Figure 2). Univariate and multivariate survival analyses demonstrated that miR-223-3p expression was an independent prognostic factor for OS in ccRCC patients (p = 0.000) (Table 2).

**Table 2:** Univariate and multivariate analyses of miR-223-3p and SLC4A4 mRNA level and patient overall survival

	Univariate analysis			Multivariate analysis^c^		
Variable	HR^a^	95%CI^b^	P	HR^a^	95% CI^b^	P
Overall survival (n = 500)						
Age (years)						
>60 (n = 249)	1.804	1.322-2.462	0.000*	1.683	1.231-2.300	0.001*
≤60 (n = 251)						
Sex						
Male (n = 327)	0.917	0.671-1.254	0.588			
Female (n = 173)						
T stage						
T3 or T4 (n = 185)	2.930	2.155-3.983	0.000*	1.628	1.137-2.333	0.008*
T1 or T2 (n = 315)						
N stage						
N1 (n = 14)	3.500	1.842-6.648	0.000*			
N0 or NX (n =486)						
M stage						
M1 (n = 77)	4.201	3.069-5.749	0.000*	2.706	1.886-3.883	0.000*
M0 or MX (n = 423)						
Grade						
G3 or G4 (n = 273)	2.602	1.840-3.679	0.000*	1.584	1.092-2.298	0.015*
G1 or G2 (n = 227)						
miR-223-3p						
High (n = 250)	2.130	1.553-2.921	0.000*	1.888	1.371-2.599	0.000*
Low (n = 250)						
SLC4A4						
High (n = 250)	0.558	0.409-0.761	0.000*	0.642	0.470-0.878	0.005*
Low (n = 250)						

a Hazard ratio, estimated from Cox proportional hazard regression model.

b Lower and upper confidence interval of the estimated HR.

c Multivariate models were adjusted for T, N, M classification, age and sex.

HR hazard ratio, CI confidential interval, T stages Tumor stage, N stage lymphatic metastasis, M stage distant metastasis

These data indicate that miR-223-3p expression is upregulated and is significantly associated with various clinicopathological parameters (such as T stage, lymphatic metastasis and distant metastasis) in ccRCC. High miR-223-3p expression predicted a poor prognosis and thus could be a potential prognostic indicator for ccRCC.

### MiR-223-3p promotes renal cancer cell proliferation, migration and invasion in vitro

**Figure 2:**
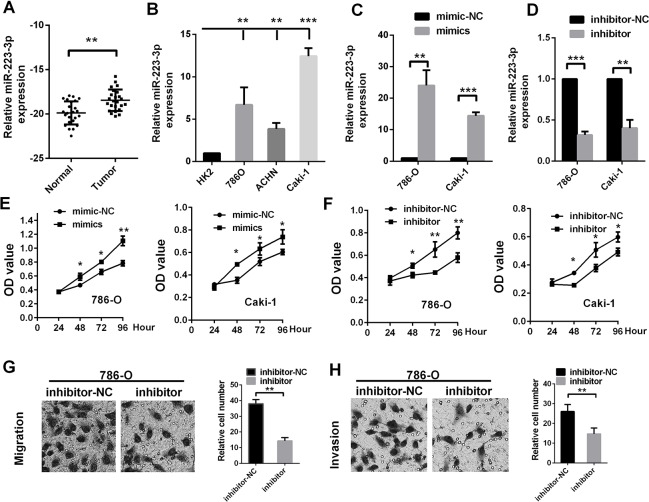
**MiR-223-3p promotes RCC cell proliferation, migration and invasion *in vitro*.** MiR-223-3p levels were measured in (**A**) renal cancer tissue samples and (**B**) cell lines. The transfection effects of (**C**) miR-223-3p mimics and (**D**) miR-223-3p inhibitors were measured in 786-O and Caki-1 cells. The effects of (**E**) miR-223-3p mimics and (**F**) miR-223-3p inhibitors on cell proliferation were measured by the CCK-8 assay in 786-O and Caki-1 cells. Representative pictures display the (**G**) migration and (**H**) invasion of 786-O cells treated with miR-223-3p inhibitors. Data are shown as the mean ± SEM. * p < 0.05; ** p < 0.01; *** p < 0.001.

We next evaluated miR-223-3p levels in ccRCC patients from The First Affiliated Hospital of Xiamen University. The results confirmed our observations in TCGA-KIRC (Figure 2A). We also determined the miR-223-3p levels of three renal cancer cell lines (A498, Caki-1 and ACHN) [27], and found that ACHN cells had the lowest expression, while Caki-1 cells had the highest (Figure 2B).

Then, we used gain- or loss-of-function assays to evaluate the effects of miR-223-3p on the proliferation, migration and invasion of 786-O (RCC) cells and Caki-1 cells. The effects of miR-223-3p mimics and inhibitors are shown in Figure 2C and 2D. MiR-223-3p overexpression significantly enhanced the proliferation of 786-O and Caki-1 cells (Figure 2E), while miR-223-3p knockdown significantly reduced their proliferation (Figure 2F). Knockdown of miR-223-3p significantly repressed cell migration and invasion compared to the negative control (NC) in Transwell assays (Figure 2G and 2H). These data indicate that miR-223-3p promotes the proliferation, migration and invasion of renal cancer cells.

### Bioinformatic analysis of miR-223-3p target genes in ccRCC

**Figure 3:**
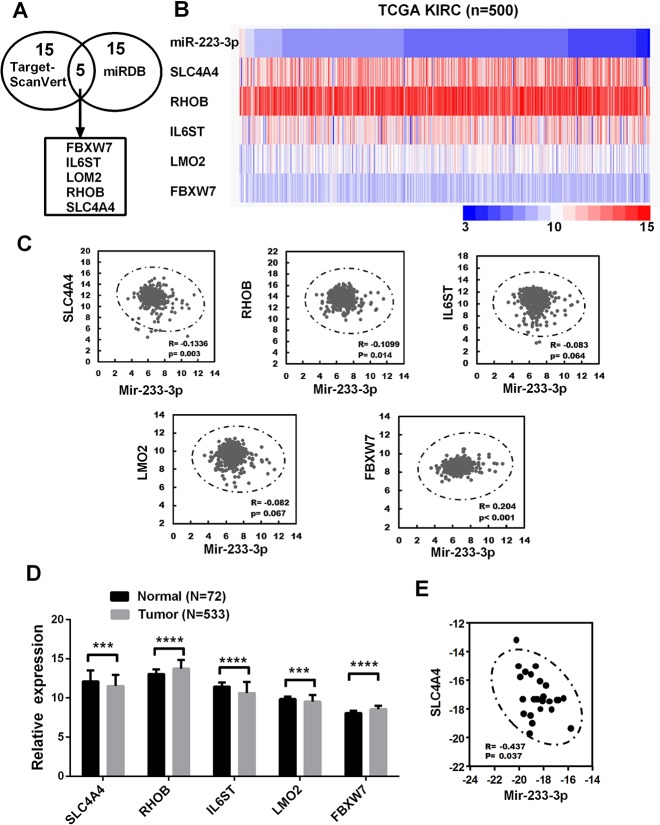
**Bioinformatic analysis of miR-223-3p target genes in ccRCC.** (**A**) Bioinformatic prediction of the top 20 mRNA targets of miR-223-3p in TargetScan and miRDB. (**B**) Heat map depicting the expression of miR-223-3p and five target genes in samples from TCGA-KIRC. (**C**) Correlation analysis of the expression of miR-223-3p and five target genes in cancer samples from TCGA-KIRC. (**D**) Relative mRNA expression of five target genes in cancer samples from TCGA-KIRC. (**E**) A qRT-PCR analysis demonstrated the negative correlation between *SLC4A4* and miR-223-3p expression in ccRCC tissues (R = -0.437, p = 0.037). Data are shown as the mean ± SEM. * p < 0.05; ** p < 0.01; *** p < 0.001.

To identify the potential targets of miR-223-3p, we used two prediction software programs (TargetScan and miRDB) to score predicted molecules. We then selected the top 20 molecules scored by each program, and identified five molecules of interest (Figure 3A): solute carrier family 4, member 4 (*SLC4A4*), Ras homolog family member B (*RHOB*), interleukin 6 signal transducer (*IL6ST*), LIM domain only 2 (*LMO2*) and F-box and WD repeat domain containing 7 (*FBXW7*). To narrow the field of prospective candidates, we performed a correlation analysis in TCGA-KIRC. A heat map revealed the correlations between miR-223-3p and the five target molecules (Figure 3B). As shown in Figure 3C, only *SLC4A4* and *RHOB* levels exhibited significant negative correlations with miR-223-3p levels. Then, we compared the mRNA levels of the five target molecules between cancerous and non-cancerous tissues in TCGA-KIRC. *SLC4A4* expression was significantly lower in ccRCC tissues than in the corresponding non-cancerous tissues, while *RHOB* expression was higher in ccRCC tissues (Figure 3D). In addition, miR-223-3p expression exhibited a significant negative correlation with *SLC4A4* expression in ccRCC patient samples from our university (Figure 3E).

### MiR-223-3p directly binds to SLC4A4

**Figure 4:**
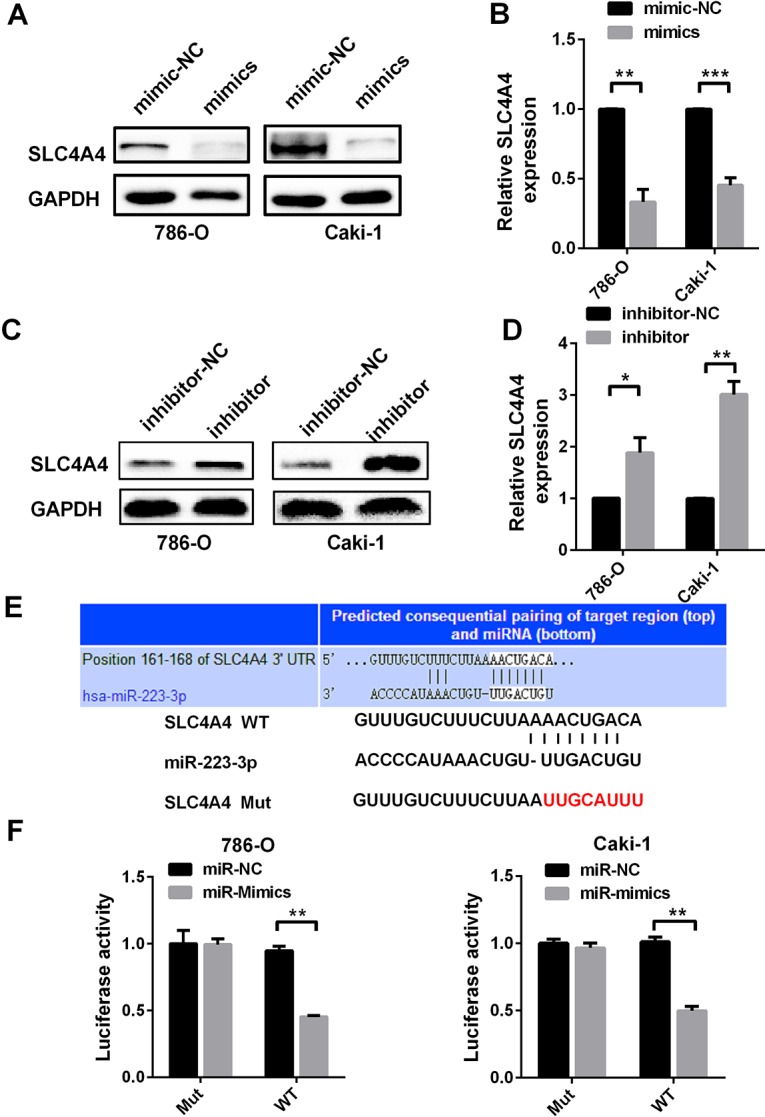
***SLC4A4* is a direct target of miR-223-3p.** (**A**) Western blotting and (**B**) qRT-PCR analysis of SLC4A4 expression in 786-O and Caki-1 cells transfected with miR-223-3p mimics versus the corresponding NC. (**C**) Western blotting and (**D**) qRT-PCR analysis of SLC4A4 expression in 786-O and Caki-1 cells transfected with miR-223-3p inhibitors versus the corresponding NC. (**E**) The predicted binding sites for miR-223-3p in the *SLC4A4* 3′-UTR. The red nucleotides are the seed-pairing target sites of miR-223-3p. (**F**) Luciferase reporter assays demonstrate that the reporter activity of 786-O and Caki-1 cells decreased by approximately 50% upon co-transfection of the wild-type *SLC4A4* 3′-UTR reporter construct and miR-223-3p mimics. Data are shown as the mean ± SEM. * p < 0.05; ** p < 0.01; *** p < 0.001.

To determine whether miR-223-3p directly binds to* SLC4A4*, we evaluated the changes in protein and mRNA levels by Western blotting and qRT-PCR, respectively, following cellular treatment with miR-223-3p mimics and inhibitors. In 786-O and Caki-1 cells, miR-223-3p mimics reduced both the protein and mRNA levels of SLC4A4 (Figure 4A and 4B), while miR-223-3p inhibitors had the opposite effects (Figure 4C and 4D).

Then, we determined the effect of miR-223-3p on the 3′-untranslated region (UTR) of *SLC4A4* using a luciferase reporter assay. Luciferase reporter constructs containing either wild-type or mutated *SLC4A4* binding sequences upstream of the firefly luciferase gene were generated (Figure 4E). Caki-1 and 786-O cells were co-transfected with the reporter vectors and mimics or mimic controls. Luciferase activity was significantly reduced after miR-223-3p mimic co-transfection with WT vectors (Figure 4F). These results suggest that *SLC4A4* is a direct target of miR-223-3p.

### SLC4A4 is significantly downregulated and associated with a poor prognosis in ccRCC patients in TCGA-KIRC

**Figure 5:**
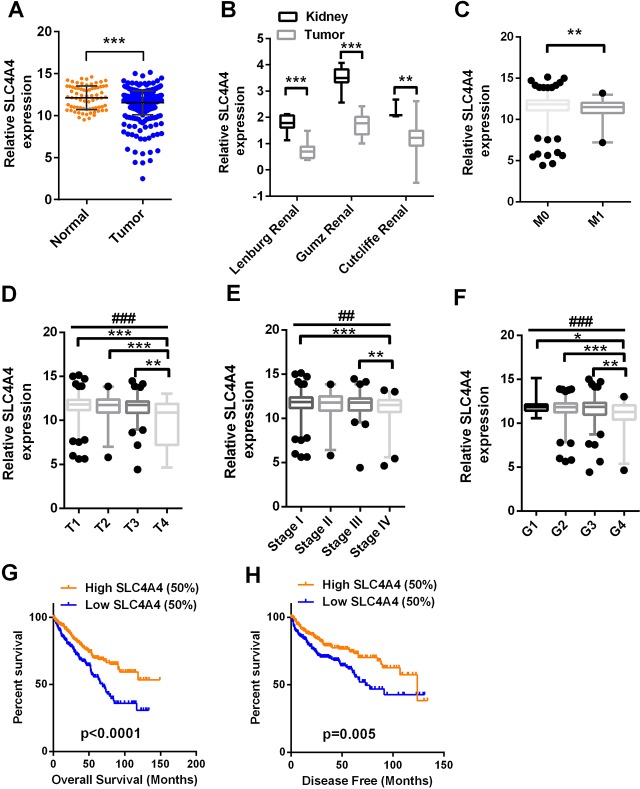
***SLC4A4* expression is downregulated in ccRCC and predicts a poor prognosis.** *SLC4A4* mRNA levels in 72 normal tissues and 533 ccRCC tissues were downloaded from the dataset of TCGA-KIRC. (**A**) *SLC4A4* mRNA levels were lower in cancer tissues than in para-cancer tissues. (**B**) SLC4A4 levels in three additional ccRCC datasets. (**C**–**H**) SLC4A4 levels were compared in ccRCC patients according to the following clinicopathological parameters: (**C**) distant metastasis, (**G**) T stage, (**E**) TNM stage, (**F**) grade, (**G**) OS and (**H**) DFS. Data are shown as the mean ± SEM. * p < 0.05; ** p < 0.01; *** p < 0.001; ## p < 0.01; ### p < 0.001.

As *SLC4A4* was found to be a direct target of miR-223-3p, we investigated *SLC4A4* mRNA levels in TCGA-KIRC. The relative expression of *SLC4A4* in log2 (FPKM+1) form ranged from 4.85 to 10.62 units in normal tissues and from 3.59 to 10.92 units in tumor tissues. The expression of *SLC4A4* was significantly lower in ccRCC tissues than in non-cancerous tissues (Figure 5A). To confirm the results from TCGA-KIRC, we examined three additional datasets in the Oncomine database (Figure 5B). Low SLC4A4 expression was detected in patients with distant metastases (Figure 5C). SLC4A4 expression was significantly lower in T stage IV than in T stages I, II and III (Figure 5D). Lower SLC4A4 levels were associated with more advanced pathological TNM stages and grades in ccRCC patients (Figure 5E and 5F). Patients with lower SLC4A4 expression exhibited shorter OS (Figure 5G, t-test, p < 0.0001) and DFS (Figure 5H, t-test, p = 0.005). Univariate and multivariate survival analyses indicated that SLC4A4 expression was an independent prognostic factor for OS and DFS in ccRCC patients (Tables 2 and 3).

**Table 3:** Univariate and multivariate analyses of miR-223-3p and SLC4A4 mRNA level and patient disease–free survival

	Univariate analysis			Multivariate analysis^c^		
Variable	HR^a ^	95%CI^b^	P	HR^a^	95% CI^b^	P
Disease–free survival (n = 407)						
Age (years)						
>60 (n = 184)	1.458	1.020-2.083	0.038*			
≤60 (n = 223)						
Sex						
Male (n = 272)	1.345	0.904-2.001	0.143			
Female (n = 135)						
T stage						
T3 or T4 (n = 140)	4.333	2.989-6.280	0.000*	2.116	1.387-3.226	0.001*
T1 or T2 (n = 267)						
N stage						
N1 (n = 12)	5.848	2.935-11.656	0.000*	2.850	1.396-5.818	0.004*
N0 or NX (n =395)						
M stage						
M1 (n = 52)	8.124	5.579-11.831	0.000*	4.901	3.222-7.455	0.000*
M0 or MX (n = 355)						
Grade						
G3 or G4 (n = 210)	3.205	2.120-4.844	0.000*	2.195	1.425-3.382	0.000*
G1 or G2 (n = 197)						
miR-223-3p						
High (n = 250)	1.342	0.938-1.921	0.108			
Low (n = 250)						
SLC4A4						
High (n = 250)	0.586	0.407-0.844	0.004*	0.607	0.419-0.880	0.008*
Low (n = 250)						

a Hazard ratio, estimated from Cox proportional hazard regression model.

b Lower and upper confidence interval of the estimated HR.

c Multivariate models were adjusted for T, N, M classification, age and sex.

HR hazard ratio, CI confidential interval, T stages Tumor stage, N stage lymphatic metastasis, M stage distant metastasis

### SLC4A4 is downregulated and involved in the biological pathogenesis of ccRCC

**Figure 6:**
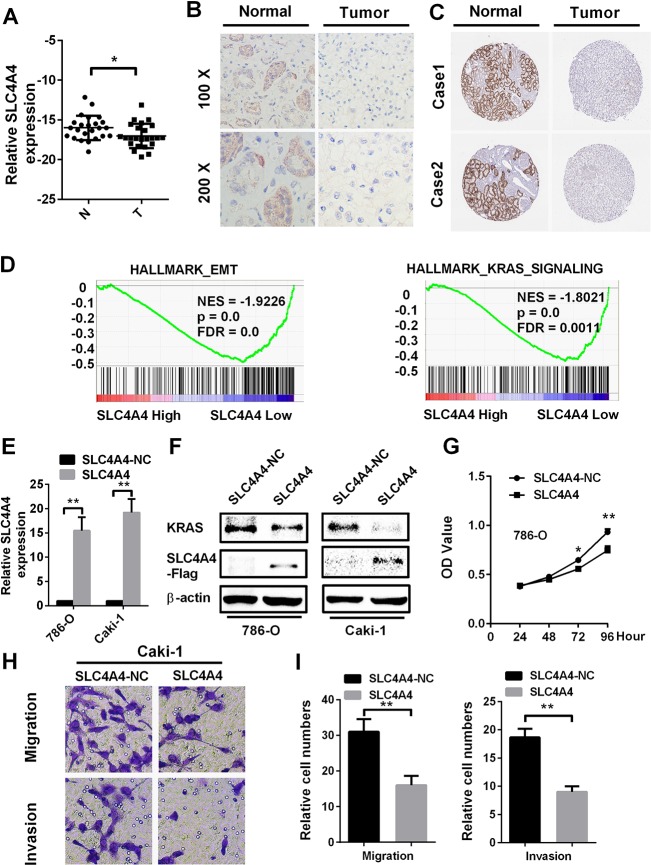
**SLC4A4 overexpression inhibits cell proliferation, migration and invasion *in vitro*.** SLC4A4 was downregulated in ccRCC tissues, as shown by (**A**) qRT-PCR and (**B**) immunohistochemistry analysis. (**C**) SLC4A4 was downregulated in ccRCC tissues, as shown by immunohistochemistry analysis from the Human Protein Atlas. (**D**) Gene set enrichment analysis comparing low and high SLC4A4 expression groups in the database of TCGA. Enrichment curves are shown for activated gene sets related to the epithelial-mesenchymal transition and KRAS signaling. (**E**) and (**F**) SLC4A4 mRNA and protein levels were successfully upregulated in 786-O and Caki-1 cells, and SLC4A4 overexpression inhibited KRAS expression. (**G**–**I**) SLC4A4 overexpression inhibited (**G**) proliferation, (**H**) migration and (**I**) invasion in Caki-1 cells. Data are shown as the mean ± SEM. * p < 0.05; ** p < 0.01; *** p < 0.001.

As *SLC4A4* was downregulated in ccRCC patients in TCGA-KIRC and was an independent prognostic factor for ccRCC, we tested SLC4A4 expression in ccRCC patient samples from our hospital by quantitative real-time polymerase chain reaction (qRT-PCR) and immunohistochemistry analyses. SLC4A4 was downregulated in ccRCC tissues compared to normal tissues (Figure 6A and 6B). Immunohistochemistry data from the Human Protein Atlas also demonstrated that SLC4A4 was downregulated in ccRCC tissues compared to normal tissues (Figure 6C). Gene set enrichment analysis demonstrated that SLC4A4 expression was associated with the epithelial-mesenchymal transition and Kirsten rat sarcoma viral oncogene (KRAS) signaling (Figure 6D).

Then, we evaluated the function of SLC4A4 in ccRCC by overexpressing SLC4A4 in RCC cell lines. The mRNA and protein levels of SLC4A4 were confirmed to be upregulated in 786-O and Caki-1 cells transfected with plasmid vectors expressing *SLC4A4* (Figure 6E and 6F). Overexpression of SLC4A4 significantly repressed the proliferation of 786-O cells compared to the NC group (Figure 6G). Furthermore, *SLC4A4* vector transfection markedly inhibited the migration and invasion of Caki-1 cells in Transwell assays (Figure 6H and 6I). These results demonstrate that SLC4A4 may function as a tumor suppressor in ccRCC cells.

### SLC4A4 attenuates the function of miR-223-3p in renal cancer cells

**Figure 7:**
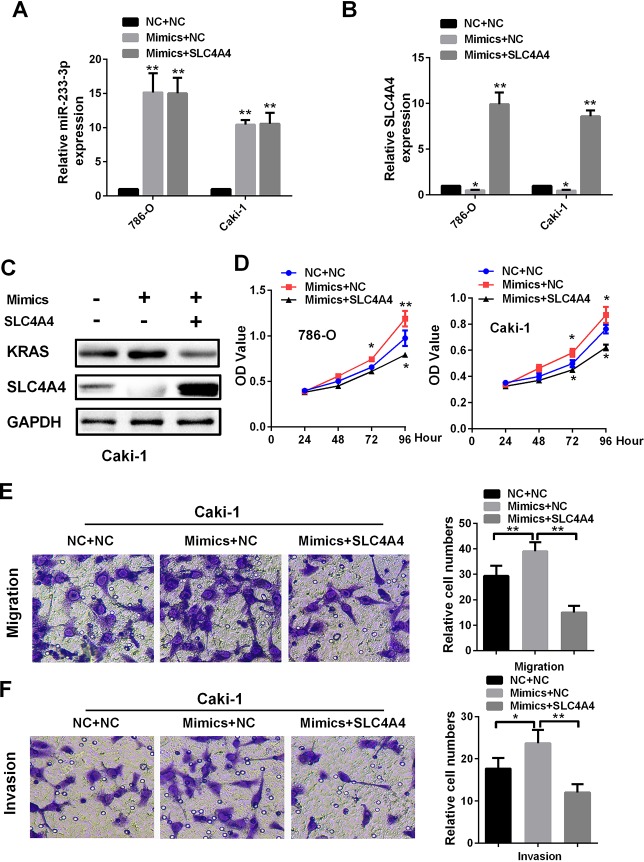
**SLC4A4 overexpression attenuates the effects of miR-223-3p mimics on cellular processes.** (**A**) MiR-223-3p and (**B**) *SLC4A4* levels were measured by qRT-PCR in 786-O and Caki-1 cells. Overexpression of SLC4A4 impaired the effects of miR-223-3p mimics on (**C**) KRAS expression, (**D**) cell proliferation, (**E**) migration and (**F**) invasion in Caki-1 cells. Data are shown as the mean ± SEM. * p < 0.05; ** p < 0.01; *** p < 0.001.

To determine whether SLC4A4 could reverse the oncogenic effects of miR-223-3p, we co-transfected renal cancer cells with miR-223-3p mimics and *SLC4A4*. The mRNA levels of miR-223-3p and *SLC4A4* are shown in Figure 7A and 7B, while the protein levels of SLC4A4 and KRAS are shown in Figure 7C. Overexpression of SLC4A4 impaired the ability of miR-223-3p mimics to induce KRAS expression (Figure 7C) and to enhance 786-O and Caki-1 cell proliferation (Figure 7D). The enhancement of Caki-1 cell migration and invasion by miR-223-3p was also reversed by co-transfection with *SLC4A4* (Figure 7E and 7F). These data demonstrate that SLC4A4 partially alleviates the oncogenic effects of miR-223-3p in RCC.

## DISCUSSION

Aberrant miRNA expression is known to be associated with cancer cell proliferation, invasion and metastasis, and miR-223-3p expression is dysregulated in many tumors. Some studies have revealed that miR-223-3p functions as an anti-tumor gene by inhibiting the metastasis and progression of osteosarcoma [28] and glioblastoma [29]. However, other studies have indicated that miR-223-3p is an oncomiRNA that is upregulated in lung cancer, gastric carcinoma and prostate cancer tissues compared with the corresponding non-cancerous tissues [24–26]. However, the expression and function of miR-223-3p in ccRCC remained unknown.

In this study, miR-223-3p was found to be significantly upregulated and associated with various clinicopathological parameters in ccRCC. Higher miR-223-3p expression was associated with worse OS and DFS in ccRCC patients. Moreover, miR-223-3p expression was greater in renal cancer cells than in normal renal cells. Overexpression of miR-223-3p promoted the proliferation and motility of ccRCC cells. Thus, we identified miR-223-3p as a key oncomiRNA in renal cancer.

Then, we searched for candidate genes using two publicly available algorithms (miRDB and TargetScan), and identified five potential target genes of miR-223-3p. Only *SLC4A4* and *RHOB* expression correlated negatively with miR-223-3p expression. However, in TCGA-KIRC, *SLC4A4* expression was significantly lower in ccRCC tissues than in non-cancerous tissues, while *RHOB* expression was higher in ccRCC tissues. Moreover, clinical data analysis revealed a negative association between miR-223-3p and *SLC4A4* levels in human ccRCC samples.

The kidney expresses the solute carrier (SLC) transporters at higher levels than any other organ [30]. SLC-family proteins have different effects on cancer development, depending on the substrates they transport [31–33]. SLC4A4 has been reported to promote colon and breast cancer growth and migration. *SLC4A4* mRNA expression depends exclusively on hypoxia inducible factor 1 subunit alpha [34], a tumor suppressor gene in ccRCC [35]. However, the function of SLC4A4 and the miRNAs regulating its expression in ccRCC have not been previously investigated.

We analyzed the expression and prognostic effects of *SLC4A4* in the database of TCGA-KIRC. *SLC4A4* was significantly downregulated in ccRCC tissues, and low SLC4A4 expression was associated with a poor prognosis. Gene set enrichment analysis demonstrated that the epithelial-mesenchymal transition and KRAS signaling were significantly enriched in response to low SLC4A4 expression in patients with ccRCC. SLC4A4 overexpression impaired RCC cell proliferation, migration and invasion *in vitro.* Furthermore, forced expression of SLC4A4 reversed the effects of miR-223-3p on cell growth and migration in Cell Counting kit-8 (CCK-8) and Transwell assays by suppressing KRAS signaling.

In the present study, miR-223-3p and *SLC4A4* were identified as important independent biomarkers that could be used to predict the clinical outcomes of ccRCC. MiR-223-3p promoted cell invasion, migration, growth and proliferation in RCC by directly binding to *SLC4A4*. This is the first report indicating that *SLC4A4* is a potential target gene of miR-223-3p in ccRCC. These results suggest that the miR-223-3p/SLC4A4 axis could be an ideal prognostic predictor and therapeutic candidate for human ccRCC.

## MATERIALS AND METHODS

### Patient samples

Surgical specimens were obtained from 24 patients between 2017 and 2018 at the Department of Urology of The First Affiliated Hospital of Xiamen University. Resected tissues were frozen in liquid nitrogen and stored at -80°C for further experiments. Informed consent was obtained from all patients, and the experimental procedures were approved by the Institutional Review Board of Xiamen University.

### RNA extraction and qRT-PCR

Tissue RNA was extracted with the TRIzol reagent (Thermo, Massachusetts, USA) according to the manufacturer’s instructions. The purity and concentration of each RNA sample was tested with a NanoDrop 2000 spectrophotometer (NanoDrop Technologies, Wilmington, DE, USA). A qPCR analysis was performed according to the manufacturer’s instructions (LightCycler 480II; Roche, Basel, Switzerland) with SYBR Green mix (Thermo, Massachusetts, USA). The relative levels of miR-223-3p and *SLC4A4* were calculated by: 2^-ΔCt^ (ΔCt = Ct_gene_–Ct_normalizer_). MiRNA primers were purchased from RiboBio (Guangzhou, China). The primers for *SLC4A4* and *GAPDH* were purchased from GENEWIZ (GENEWIZ, Suzhou, China): *SLC4A4*, Forward 5’-TTCACGGAACTGGATGAGCT-3’, Reverse 5’-ACTGTGGGAGAGAAGAAGCC-3’; *GAPDH*, Forward 5’-GAGTCAACGGATTTGGTCGT-3’, Reverse 5’-GACAAGCTTCCCGTTCTCAG-3’.

### Cell culture and transient transfection

HK2, 786-O, ACHN and Caki-1 cells were purchased from The American Type Culture Collection (USA). Cancer cells were cultivated in high-glucose Dulbecco’s modified Eagle’s medium (DMEM; Wuhan Boster Biological Technology, Ltd., Wuhan, China) containing 10% fetal bovine serum (Gibco; Thermo Fisher Scientific, Inc., Waltham, MA, USA) at 37°C in a 5% CO_2_ incubator, as previously reported [36]. Human miR-223-3p mimics, miR-223-3p inhibitors and the corresponding controls were synthesized by RiboBio. Plasmid vectors expressing *SLC4A4* or the NC were constructed by Genechem (Shanghai, China). Lipofectamine 2000 reagent (Invitrogen, Carlsbad, CA, USA) was used to transfect cells with miR-223-3p mimics, miR-223-3p inhibitors, the *SLC4A4* plasmid and the corresponding controls in six-well plates according to the recommendations of the manufacturer.

### Cell proliferation analysis

Caki-1 and 786-O cells were first transfected with miR-223-3p mimics, miR-223-3p inhibitors, *SLC4A4* or the NC. Then, the cells were added to a 96-well plate at a density of 3x10^3^ cells/well. The cell proliferation rate (optical density value) was determined with a CCK-8 assay (Dojindo Molecular Technologies, Inc, Rockville, MD, USA) according to the manufacturer’s protocol. Two hours after the addition of 10 µL of CCK-8 solution to each well, the optical density value was measured at 450 nm. Three independent experiments were conducted for each assay.

### Migration and invasion assays

Migration assays were performed as previously described [37]. Cells were homogenized in the absence of plasma for 24 h, and then cultured at 1x10^5^ cells/well in 24-well Transwell plates with polycarbonate membrane inserts (Corning Inc., Corning, NY, USA). For the invasion assays, the membrane was coated with Matrigel (Thermo Fisher Scientific), and cells were cultured at 2x10^5^ cells/well. After incubation for 24 hours, the cells were fixed with 100% methanol and stained with 0.05% crystal violet. Five random fields were counted, and three independent experiments were conducted for each assay.

### Luciferase assays

Wild-type and mutant *SLC4A4* 3′-UTR reporters were purchased from RiboBio. Tumor cells transfected with miR-223-3p mimics or miR-NC in 24-well plates were co-transfected with 500 ng of a luciferase reporter with Lipofectamine 2000 reagent (Thermo Fisher Scientific). All dual luciferase assays were performed with a Dual Luciferase Assay kit (Promega, Madison, WI, USA). Normalized Renilla-luciferase values were calculated relative to the control, according to the manufacturer’s protocol.

### Western blotting

Tissues and cells were pyrolysed in a protein lysis system containing radioimmunoprecipitation assay buffer (Wuhan Boster Biological Technology, Ltd.), a protease inhibitor cocktail (Roche Diagnostics, Indianapolis, IN, USA) and PMSF (Wuhan Boster Biological Technology, Ltd.). Protein concentrations were measured with a bicinchoninic acid kit (Beyotime Institute of Biotechnology) at 562 nm. Thirty micrograms of each protein were subjected to sodium dodecyl sulfate polyacrylamide gel electrophoresis and transferred to polyvinylidene fluoride membranes (EMD Millipore, Bedford, MA, USA) for 90 min. After the transfer, the membranes were blocked in phosphate-buffered saline with 5% non-fat milk for one hour and then incubated with antibodies against SLC4A4 (1:1000; A5332; ABclonal Biotech Co., Ltd.), KRAS (1:1000; A11059; ABclonal Biotech Co., Ltd.) and GAPDH (1:2000; BM3876; Wuhan Boster Biological Technology, Ltd.) at 4°C overnight. The next day, the membranes were washed and then incubated with secondary antibodies (1:5000; BA1020; Wuhan Boster Biological Technology, Ltd.) at room temperature (25°C) for two hours. Finally, the membranes were washed, and protein levels were detected with a ChemiDoc-XRS+ (Bio-Rad Laboratories, Inc., Hercules, CA, USA).

### Immunohistochemistry

Adjacent normal tissues and ccRCC tissues were fixed in formalin, dehydrated and embedded. Tissue sections were then incubated with a primary rabbit SLC4A4 polyclonal antibody (1:100) at 4°C overnight. The sections were washed three times with phosphate-buffered saline and then incubated with a goat anti-rabbit secondary antibody (1:200; GB23303; Servicebio, Inc., Woburn, MA, USA) for two hours at room temperature.

### Bioinformatic analysis

RNA-seq data from ccRCC patients (TCGA-KIRC) and the patients’ clinical information were obtained from the Xena Functional Genomics Explorer (https://xenabrowser.net/heatmap/) of the University of California Santa Cruz [38] and the Oncomine database (https://www.oncomine.org). Detailed clinical information on staging can be found at (https://www.cancer.org/cancer/kidney-cancer/detection-diagnosis-staging/staging.html). Protein immunohistochemistry data were obtained from the Human Protein Atlas (https://www.proteinatlas.org/). Candidate miR-223-3p target genes were identified through two publicly available algorithms, miRDB (http://mirdb.org/miRDB) and TargetScan (http://www.targetscan.org). Gene set enrichment analysis was used to determine the SLC4A4 pathway involved in the pathogenesis of ccRCC in patients from TCGA-KIRC (http://www.broadinstitute.org/gsea) [39]. Following the performance of 1,000 permutations, p values < 0.05 and a false discovery rate < 25% were required for the enriched gene set analysis to be considered significantly enriched.

### Statistical analysis

The RNA levels of paired samples were analyzed with a paired-sample t-test, while those of unpaired samples were analyzed by one-way ANOVA for four groups (#) or by the t-test for two groups (*). The AUC and ROC curve were used to distinguish the clinical classifications. A Kaplan-Meier curve was used to evaluate the survival rate according to miR-223-3p and SLC4A4 expression with a log-rank test. Long and short OS and DFS were discriminated based on good (≥ 5 years alive or disease-free) and poor (death or recurrence or progression in ≤ 2 years) prognoses [40]. The prognostic significance of miR-223-3p and SLC4A4 in ccRCC were analyzed by univariate and multivariate Cox proportional hazard regression analyses. P values < 0.05 were considered to be statistically significant. All statistical analyses were conducted with SPSS Statistics 22.0 (IBM SPSS, Chicago, IL, USA).

## SUPPLEMENTARY MATERIALS

Supplementary Figures
