# Supplementary material for: MiR-223-3p promotes cell proliferation and metastasis by downregulating SLC4A4 in clear cell renal cell carcinoma
Source: Aging (Albany NY). 2019 Jan 22;11(2):615–33. doi: 10.18632/aging.101763 (PMC6366987; doi:10.18632/aging.101763)
Supplement: Supplementary Figures [file aging-11-101763-s001.pdf]

SUPPLEMENTARY MATERIAL

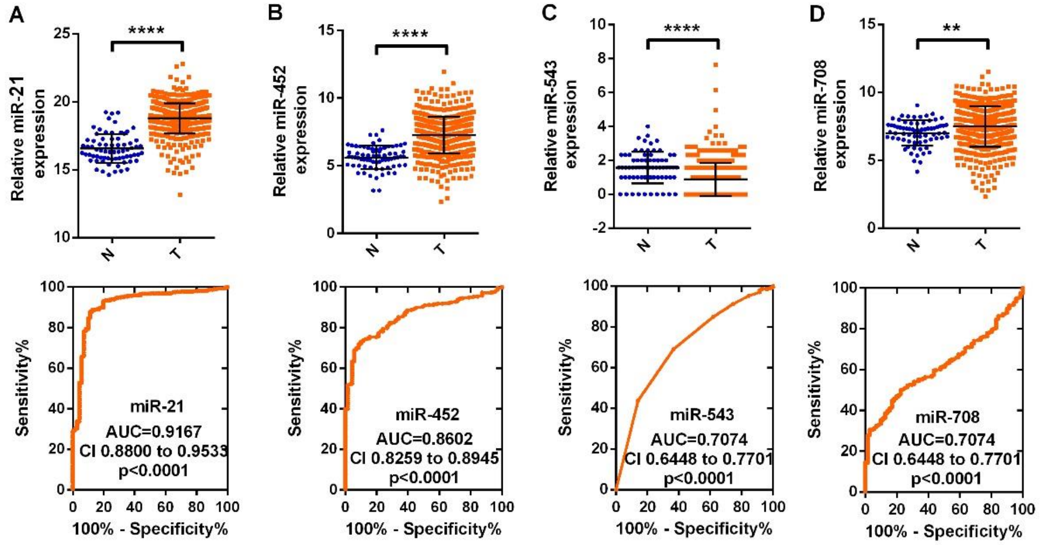

Supplementary Figure 1. External comparison of miRNAs expressions in the Cancer Genome Atlas ccRCC dataset and AUC. (**A**) for miR-21 and (**B**) for miR-452, (**C**) for miR-543 and (**D**) for miR-708. ** P < 0.01; *** P < 0.001, **** P < 0.0001.

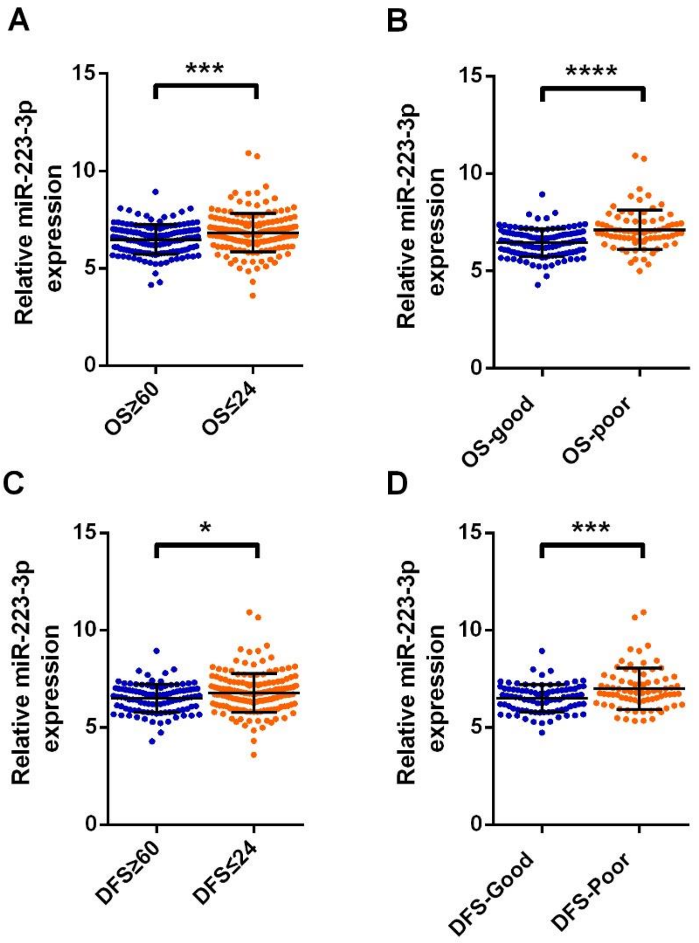

Supplementary Figure 2. The correlation between miR-223-3p expression and long and short OS and DFS of ccRCC patients. MiRNA levels between patients with good and poor prognosis were compared by independent sample t-test. (**A**) for long and short OS, (**B**) for good and poor prognosis of OS, (**C**) for long and short DFS, (**D**) for good and poor prognosis of DFS. * P < 0.05, ** P < 0.01; *** P < 0.001, **** P < 0.0001.
